# Notch Signaling in HSC Emergence: When, Why and How

**DOI:** 10.3390/cells11030358

**Published:** 2022-01-21

**Authors:** Roshana Thambyrajah, Anna Bigas

**Affiliations:** 1 Program in Cancer Research, Institut Hospital del Mar d’Investigacions Mèdiques, CIBERONC, 08003 Barcelona, Spain; 2 Josep Carreras Leukemia Research Institute, 08003 Barcelona, Spain

**Keywords:** Notch signaling, HSC, developmental hematopoiesis, AGM

## Abstract

The hematopoietic stem cell (HSC) sustains blood homeostasis throughout life in vertebrates. During embryonic development, HSCs emerge from the aorta-gonads and mesonephros (AGM) region along with hematopoietic progenitors within hematopoietic clusters which are found in the dorsal aorta, the main arterial vessel. Notch signaling, which is essential for arterial specification of the aorta, is also crucial in hematopoietic development and HSC activity. In this review, we will present and discuss the evidence that we have for Notch activity in hematopoietic cell fate specification and the crosstalk with the endothelial and arterial lineage. The core hematopoietic program is conserved across vertebrates and here we review studies conducted using different models of vertebrate hematopoiesis, including zebrafish, mouse and in vitro differentiated Embryonic stem cells. To fulfill the goal of engineering HSCs in vitro, we need to understand the molecular processes that modulate Notch signaling during HSC emergence in a temporal and spatial context. Here, we review relevant contributions from different model systems that are required to specify precursors of HSC and HSC activity through Notch interactions at different stages of development.

## 1. Introduction to HSC Development

Hematopoietic stem cells (HSCs) sustain the adult blood system by generating mature blood cells of all lineages through multi-potent progenitors of intermediate stages [1]. During embryogenesis, the hematopoietic system is established through several waves starting from Embryonic day (E) 7.5. In mouse, the earliest blood cells are produced in the blood islands of the yolk sac (extra embryonic) which continue to distribute hematopoietic cells with erythro-myeloid lineage potential by E8.5 and multipotent hematopoietic cells also with lymphoid lineage potential at later stages [2,3,4]. Cells originating from the early waves of hematopoiesis also include tissue resident macrophages that infiltrate various organs and fulfil tissue-specific and niche-specific functions, including functions during HSC development [5,6]. However, the first HSCs with hematopoietic reconstitution capacity are detected from E10.5 onwards within the embryo (intra-embryonically). They are particularly enriched in the trunk of the embryo where the aorta, gonads and the mesonephros meet (AGM). The hematopoietic stem and progenitor cells (HSPC) accumulate as Intra-aortic hematopoietic clusters (IAHC) in the dorsal aorta (DA) [7,8,9,10,11]. Although nascent HSCs have been associated to other sites (umbilical cord, placenta, head [12,13,14,15]), blood emergence is closely associated with a specialized endothelial cell population, termed hemogenic endothelium (HE), that trans differentiate to blood by losing their endothelial identity and gaining hematopoietic potential. Over the years, several studies have conclusively demonstrated this endothelial-to-hematopoietic transition (EHT) by in vivo imaging of different animal models, as well as in vitro differentiation to blood from Embryonic Stem (ES) cells [16,17,18,19,20,21,22]. HE cells can be identified by the co-expression of endothelial marker gene expression such as CD31, CDH5, ACE and CD44 and key hematopoietic transcription factors, including Runx1, Gfi1 and Gata2 [23,24,25,26,27,28,29,30]. The earliest transcription factors detected in the HE, Runx1 and Gata2, are downstream of Notch signaling, [31,32] and later the expression of Gfi1 is detected in a discrete subset of Runx1 positive cells that are restricted to the HE and IAHC, while runx1 expression extends to the subaortic mesenchyme [30]. Several other surface markers and transcription factors have been described to enrich HSC activity, including Sca1, Gpr56, CD27 and CD201 (PROCR) [33] [34,35,36,37]. Once the EHT process is completed, the cells proliferate and recruit other cells [29,38] forming IAHC that appear associated to the ventral wall of the dorsal aorta starting between the embryonic days E10.25–E12 in the mouse (week 4–5 in human embryo). Although HE and IAHC can be observed on both the ventral and the dorsal side of the aorta within this time window, only the IAHC associated with the ventral side contain transplantable HSCs [27,39]. This has been mainly attributed to pathways, including BMP, hedgehog and Notch signaling that are polarized to the ventral domain [39]. The emerging HSPCs then migrate to the fetal liver for maturation and expansion [40]. The sites of HSC emergence and their migration between hematopoietic niches are very well conserved in vertebrates [41]. In addition, in the zebrafish, HSPCs emerge from the dorsal aorta of the trunk. However, unlike in the mouse model, the early erythroid-myeloid progenitors and the emergence of progenitors with HSC properties occur within a shared spatial and temporal manner [42]. At least in the mouse, transplantation assays performed at different time points of HSPC emergence, early (pre)-HSC can readily contribute to the blood system of neonates, but not directly to the adult system [43]. This potency is only evident in HSCs that are older than E11.5. Even then, only a very small fraction of these HSPCs are functional HSCs [25,26,27,44], with the majority being blood progenitors. Therefore, although there is consensus regarding the site of HSC emergence. It is unclear whether HPCs and HSC share the same HE precursors, or if in fact, the HE is a heterogeneous cell population with different capacities. Moreover, further clarity is required in understanding which molecular pathways are unique to HSC emergence or shared with HPCs. Adding further to the complexity, EHT and HSPC emergence occurs at a developmental stage when angiogenesis is in progress and vascular identity (arterial versus venous) is being established. Therefore, it is highly plausible that both these processes share common signaling pathways to some extent.

Importantly, arterial fate acquisition and HSC emergence are dependent on Notch activity, but its specific requirement in HE and EHT is only now to emerge. There is some evidence suggesting that the arterial fate can be uncoupled from HE, but also evidence arguing that arterial specification of the aorta is a prerequisite for HE specification and subsequent EHT of HSPC/HSCs. Currently, these questions remain open. In this review, we will discuss and highlight the current studies that can give us clues on the requirement of Notch activity for HE and specifically, HSC emergence in mouse and zebrafish since these two model organisms have contributed the most to our understanding of embryonic hematopoiesis.

## 2. The Basics of Notch Signaling

**Figure 1:**
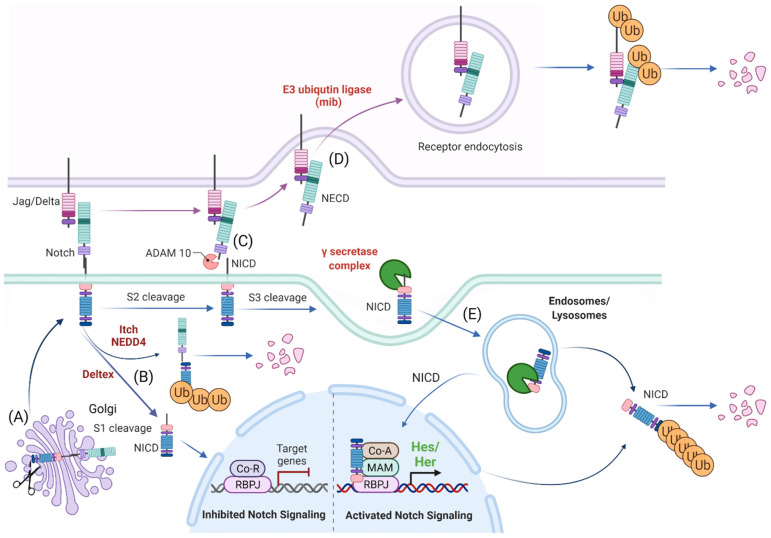
Schematic representation of Notch signaling molecule interaction, their localization within the cellular compartment and their life cycle. (**A**) Notch receptors undergo S1 cleavage in the Golgi vesicle. (**B**) Notch receptors can be directly degraded (without ligand interaction) via NEDD4/ITCH from the cellular surface or processed to Notch Intracellular Domain (NICD) in the absence of a Notch ligand by Deltex. (**C**) In the canonical Notch receptor processing, Notch receptor is cleaved upon contact with a ligand by ADAM family members at the c-terminal (intracellular) end that leads to exposure of the S3 cleavage site. (**D**) The ligand sending cell endocytoses the extracellular domain of the Notch receptor (NECD) that it “pulled off” when interacting with the receptor. (**E**) The NICD is then further processed by the γ-secretase complex to an activated NICD (cleavage at Val1744) within endosomes/lysosomes that can either be targeted for degradation or translocate to the nucleus for target gene modulation. Ub: ubiquitination, Co-R: undefined co-repressor, Co-A: undefined co-activator, MAM: MAML. Created with BioRender.com (accessed on 15 January 2022).

**Figure 2:**
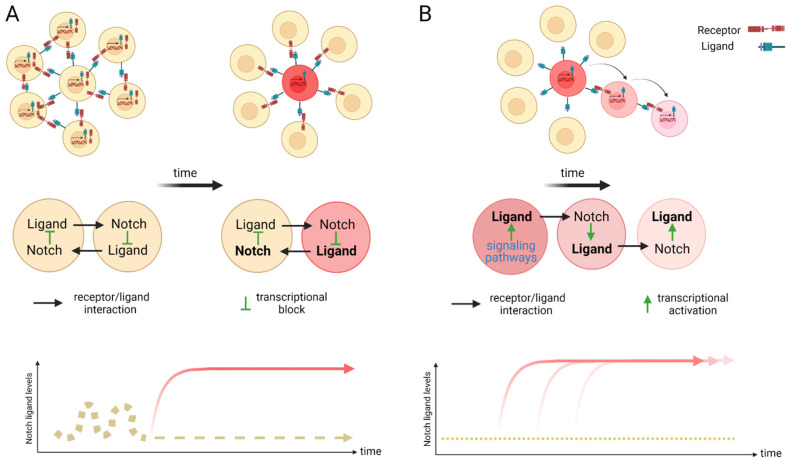
Scheme of lateral inhibition (**A**) and lateral induction (**B**). (**A**) Within an equipotent cell population with fluctuating Notch activity, a stochastic up-regulation of Notch activation can induce the expression of the Notch downstream transcriptional repressors that in turn silence the expression of ligand transcription. The remaining receptors on the surface of this cell can now act as signal senders for neighboring cells and induce a different fate to its own. Over time, a salt and pepper pattern emerges. A cell with high levels of ligand (sender) is positioned surrounded by receptor expressing cells (receivers). This mechanism of cell fate determination is termed lateral inhibition. (**B**) Lateral induction is the term used for sequential induction of Notch activity within adjacent cells. In this scenario, Notch activity induces further transcriptional activation of Notch receptors and ligands. The cell stays activated (through the newly synthesized receptor) but further activates the adjacent through the ligand. This cycle is repeated over time to establish a group of cells with the identical cell fate. Arrow depicts direction of activation. Created with BioRender.com (accessed on 15 January 2022).

The Notch signaling pathway controls cell fate decisions during embryonic development and is highly conserved in metazoan [45]. In the classical model, Notch signaling is established through cell–cell contact. Adjacent cells express the Notch receptor and/or ligand on their cell surface. Upon interaction, the receptor is activated and results in the nuclear translocation of the active form of the Notch signaling molecule (Figure 1). In mammals, there are four Notch receptors (Notch1–4) and five ligands: there are three Delta ligands (Dll1, Dll3 and Dll4) and two Jagged ligands (Jag1 and Jag2), but in zebrafish some of these receptors and ligands have undergone duplications and therefore have two isoforms [42]. Typically, upon ligand binding (Delta or Jagged), the Notch receptor undergoes three protease cleavages thereby releasing the Notch Intracellular Domain (NICD), which then translocates into the nucleus. In the nucleus, the NICD forms a complex with its coregulator, RBPJ (Recombination signal-Binding Protein for Ig Kappa J region) and recruits co-activators such as MAML (Mastermind-Like) to its gene targets. The best characterized NICD–RBPJ complex targets are the transcriptional repressors genes of the Hes/Her family in vertebrates (Figure 1) [46]. These Hes/Her transcription factors can repress genes driving cell specification, cell differentiation and cell cycle arrest [46]. Hes/Her can also form a negative feedback loop and repress Notch ligand expression in a particular cell. This first receiver can now send out a signal and inhibit the neighboring cell through the remaining ligands on its cell surface (Figure 2). This lateral inhibition mechanism results in one cell that is unique in a homogenous cell population. This cell can be destined to acquire a distinctive fate, by inducing Notch activation and repressing this specific fate in the neighboring cells (Figure 2) [47,48,49]. However, the reverse mechanism can be used to specify a small group of cells with the identical fate. Here, an adjacent cell expresses the Notch ligand upon Notch activation which then further spreads this (positive) feedback loop (lateral induction) to the next neighboring cell [50,51]. Subsequently, both the interacting cells and a small group of cells within a population acquire the same fate (Figure 2). On the contrary, Notch receptors and ligands can also form cis interactions that inhibit or activate Notch signaling. In these instances, the receptor and the ligand are present on the surface of the same cell, form a complex and thereby mask this cell from further Notch activity [52,53,54]. In vertebrates, different combinations of Notch receptor and ligand can be expressed in a cell population and their spatiotemporal abundance contributes further to the complexity of Notch signaling. Interactions of different ligands with the same receptor can also trigger distinct responses. For example, during angiogenesis, JAG1 and DLL4 drive different outcomes in the control of cell fate decisions. Similarly, Dll1 and Dll4 induce different Notch activation dynamics resulting in opposing gene programs and cell fates [55]. Finally, Fringe glycotransferases (Radical, Lunatic and Manic) can modify the Notch receptor and alter the affinity between Notch receptors and their ligands and further fine tune the cellular Notch activity [56].

## 3. Processing of the Notch Receptors and Ligands

### 3.1. Release of the Transcriptionally Active NICD

The Notch receptor is cleaved at multiple sites and stages before its NICD is released into the nucleus. Initially, the Notch receptor is post-translationally cleaved at the S1 site whilst in the trans-Golgi network by a Furin-like protease, which results in a heterodimer that is held together by Ca^2+^-dependent ionic bonds and can be integrated into the cell membrane. Here, it is important to note, that this first cleavage exposes the negative regulatory region (NRR) at the base of the extracellular domain (ECD) and this is critical in preventing Notch activation in the absence of the correct signals. When a receptor–ligand interaction occurs, conformational changes in the NRR allow access to the ADAM proteins to the S2 cleavage site that is also based in the ECD of the Notch receptor [57,58,59]. Once the S2 cleavage occurs, further conformational changes expose the S3 cleavage site on the intracellular domain of the receptor to the γ-secretase complex that is comprised of multiple protein subunits including Nicastrin, Anterior pharynx defective-1, Presenilin enhancer-2 and the catalytically active subunit PRESENILIN. This complex is present at the plasma membrane and localizes to early/late endosomes and in lysosomes. Notch receptor and γ-secretase co-localization to the endocytic compartment is critical to Notch activation. During endocytosis, CATHRIN is recruited to the plasma membrane and adheres to lipid- or protein-binding domains at the membrane with the help of adaptor proteins. These adaptors help to form a curved vesicle called CLATHRIN-coated pits. These pits invaginate with the help of bending-proteins, such as EPSIN and form vesicles that eventually bud off from the membrane. The vesicle is then uncoated and can then fuse with other intracellular structures such as endosomes and lysosomes [60,61]. The S3 cleavage occurs within these intracellular vesicles and releases the NICD and allows Notch signaling to be initiated.

### 3.2. The Role of Notch Ligand in Activating Notch Signaling

Notch activation is not only dependent on receptor-ligand interactions, but also on the ECD dissociation from the receptor and its trans-endocytosis into the ligand-expressing cell [62,63]. It has been suggested that this pulling force is necessary for conformational changes in the NRR region and for S2 cleavage by ADAM family members, ADAM10 and ADAM17/TACE, after which the ECD is free to be trans-endocytosed [63].

### 3.3. Notch Ligand Independent NICD Activation

As an alternative, Notch endocytosis has been suggested not only as necessary for its activation, but also in order to decrease the level of Notch signal by reducing its expression on the cell surface. Notch can indeed be marked for degradation via ubiquitination by E3-ligases such as AIP4/ITCH [64] or Nedd4 [65,66]. It is then endocytosed via NUMB which recruits the AP2-clathrin adaptor-complex [67,68]. Finally, Notch activity can also occur independent of ligand interaction. DELTEX, a E3 ubiquitin ligase, can facilitate the processing of the Notch receptor in the endosomes and thereby release the NICD in the absence of a ligand [69,70].

In conclusion, complete processing of Notch requires multiple cleavages, classically through interaction with a ligand and internalization of the receptor, where it then becomes cleaved and fully activated in the endosome. Each of these steps requires the participation of several proteins and they all together fine tune the final NICD activity.

## 4. Notch Activity during Zebrafish Angiogenesis

Fate-mapping studies in zebrafish of mesodermal progenitors labeled at midblastula or gastrula stages indicate that at least some of the putative hemangioblast are bipotential and capable of giving rise to cells of both lineages, although the majority of labeled cells give rise to only one lineage [71,72,73]. Nevertheless, the early angioblasts assemble via migration to the midline of the trunk into two axial vessels: the dorsal aorta and the posterior cardinal vein (PCV), found just below the DA. Interestingly, fate-mapping studies in the zebrafish indicate that angioblasts become restricted to either an arterial or venous fate before they start to migrate to the trunk midline to form the DA and PCV [74]. Hedgehog (Hh) is a morphogen known to regulate epithelial/mesenchymal interactions during embryonic development and sits at the apex of the signaling cascade that leads to arterial and venous identity [74,75]. In zebrafish, its secreted from the endoderm during gastrulation and later from the notochord and forms a decreasing gradient to induce the expression of two families of angiogenic cytokines, *vascular endothelial growth factor-1* (*vegf*) from the somites and *angiopoietins-1* and *-2* (Ang1/Ang2) [76]. Vegf-A is produced by multiple cell types, including somites and smooth muscle cells and regulates differentiation, proliferation and survival of ECs [77,78]. In angioblasts that have arterial identity, Vegf interacts with the Vegf receptor *Flk1/Vegfr2* and *Np1* complex to induce the activation of the Notch and Erk signaling pathways [79,80]. In contrast, in venous angioblasts, *chicken ovalbumin upstream promoter transcription factor II* (Coup-TFII) acts to suppress the Notch and Erk pathways and thereby repress arterial fate induction [74,80]. The Notch pathway activation is required for imperative for arterial specification and arterial marker expression, including Ephrinb2, Notch1, Notch4, Dll4 and the Notch downstream target gene Hey2 (a hairy/enhancer-of-split-related basic helix-loop-helix transcription factor) [81]. The venous angioblast express COUP-TFII and the B4 ephrin receptor (EphB4) [80,82].

## 5. Notch Activity Contribution to Zebrafish HSPC Emergence from the Dorsal Aorta

Analysis of the *mind bomb* mutant in zebrafish embryos has demonstrated the requirement of Notch signaling in HSPC emergence from the dorsal aorta. *Mind bomb* is an E3 ubiquitin ligase, which is essential for endocytosis of Notch ligands upon Notch receptor and ligand interaction [83]. The mutants display a complete absence of transcripts for the HSC marker genes *cmyb* and *runx1* in the ventral floor of the dorsal aorta at 36 h post fertilization (hpf), although the erythro-myeloid progenitor (EMP) generation is unaffected [32,84]. Accordingly, overexpression of the Notch intracellular domain resulted in an expansion of *cmyb* and *runx1* at 36 hpf that reaches into the venous endothelium, but notably, without increasing *efnB2a* expression into the vein [32]. Studies have also addressed the requirement for Notch signaling in HSPC emergence by generating morphants for Notch receptors and ligands. The two zebrafish Notch ligands *dlc* and *dld* are expressed from the somitic tissue around 17.5 hpf, just after the axial vessels are beginning to segregate. Both ligands are dependent on Wnt16 expression from the somites and critical for proper blood emergence [85]. Enforced expression of Nicd at 14 hpf could rescue *cmyb* expression at 36 hpf in these morphants. However, later induction failed to induce *cmyb* expression along the dorsal aorta [85]. The authors reason that the critical phase WNT16 (WNT family member 16) mediated Notch signaling required for HSC specification occurs between 15–17 hpf and not thereafter. A later study from the same group identified the adhesion molecules Jam1a and Jam2a as the critical contributors to this *dlc* and *dld* ligand function. *jam1a* and *jam2a* are expressed on early vascular progenitors and are activated through Notch by the *dlc* and *dld* ligand presenting somites [86]. When this interaction is missing, arterial specification is unaffected, but HE is lost [86]. In order to identify if any of the Notch signaling molecules were affected by Jam1a loss, they sampled all the aortic Notch receptor and ligand genes (*notch1a*, *notch1b*, *notch3*, *dlc* and *dll4*), but detected no changes. In yet a different study, the same group discovered *notch3* receptor expression in the somites and on the vascular progenitors at early time points. By using morpholino mediated knock down approaches, they show that Notch3 is molecularly situated downstream of *dlc* and *dld* [87] that is important for HE specification, but dispensable for arterial fate determination. Only a second wave of Notch activity which is driven by the aortic expression of Notch1a and Notch1b is crucial for both arterial identity and most importantly, HSPC formation [87]. Which Notch receptors the zebrafish Dlc and Dld interact with and which downstream targets they mediate is currently, not known. At least within the dorsal aorta, where the two Notch isoforms *notch1a* and *notch1b* are expressed, the ligand Jag1a seem to play a dominant role in HSPC emergence, as in the mouse. Morphants for Jag1a display an established arterial fate but have compromised HE and HSPC formation ([88]. Finally, time lapse imaging on a Notch responsive transgenic zebrafish line illustrates Notch activity in HE and cells undergoing EHT which decreases as they leave the DA [89]. 

Altogether, Notch activity is pivotal for HSPC emergence from the DA of the zebrafish model. Two waves of Notch activity seem required. At least in the zebrafish model, firstly, Notch3 from the somites interacts with the ligands Dlc and Dld in the vascular progenitors. Epistatic analyses demonstrate that Notch3 function lies downstream of Wnt16, which is required for HSC specification through its regulation of two Notch ligands, dlc and dld [87]. Once the vascular progenitors segregate according to their arterial/venous fate, Notch activation in aortic cells through Notch1a/Notch1b and Jag1a is essential. Together, they provide the indispensable Notch activity levels for HE specification and HSPC emergence.

### Downstream Targets of Notch Activation in Zebrafish HSPC

Genome duplication within the teleost lineage has given rise two Gata2 paralogs in zebrafish, *gata2a* and *gata2b*. Gene expression analysis demonstrates a distinct pattern of *gata2a* and *gata2b* expression in zebrafish [90]. Gata2a is expressed throughout the endothelium, but Gata2b is restricted to the HE subpopulation of the DA. Expression of Gata2b begins in the vascular cord during posterior lateral mesoderm migration and is initiated in a subpopulation of fli1a+ (early vascular progenitors) cells [90]. This study on genetic morphants demonstrates that Notch1a and Notch1b are required for *gata2b* expression in the aorta, but not *gata2a*. Furthermore, *gata2b* morphants lose *runx1/cmyb* expression in the trunk, but not the expression of EfnB2a [90] with the study further highlighting how two isoforms can be employed to direct different cell fates. Finally, another direct Notch target, Hey2 is demonstrated to act upstream of Notch activation in the dorsal aorta in a zebrafish model [81]. *Hey2* expression is first detected in the early angioblast when the formation of the axial vessels and independent of Notch signaling, as the *hey2* expression persists in Rbpj morphants and *mind bomb* mutants [81]. Intriguingly, Hey2 morphants do not express the receptor *notch1b* or *notch5* in the dorsal aorta that are important for Notch signaling in arterial cells, but the Notch ligand *dll4* is readily detected, suggesting that initiation of *dll4* expression is not dependent on Notch activation through *notch1b* (or *notch5*). Consequently, the arterial gene *efnb2a* is not expressed (due to lack of Notch1b), *flt4* is not downregulated (venous gene that is usually silenced by Notch activity) and *runx1+* hematopoietic cells do not emerge from the DA angioblast cord. However, the lack of hematopoietic progenitors can be rescued by enforced expression of NICD in Hey2 morphants, suggesting that Hey2 indeed acts upstream of Notch activity induced by Notch1b in the aorta [81]. In summary, in zebrafish, the Notch target *hey2* seems to mark the arterial and HE primed endothelial cells from an early precursor stage already. During the dorsal aorta segregation from the venous endothelium, Notch activity is resumed for arterial identity and hematopoietic commitment. Here, Notch signaling mainly fine tunes the expression and cross regulation of the transcription factors *gata2*.

## 6. Notch Activity during Mouse Angiogenesis

### 6.1. Notch during Early Angiogenic Cord Formation

The mouse angiogenic development is less well studied compared to the zebrafish model since assessment of early embryos is limited and ex vivo culture is not established. Nevertheless, the early events of vasculogenesis and angiogenesis are conserved to a high degree within vertebrates. The existence of an hemangioblast precursor has also been postulated in the mouse embryo as well as in the zebrafish, but its existence is both rare and under debate [91,92,93]. Fate mapping studies lead to the discovery that the endothelium and blood cells are already specified at the pre-gastrula epiblast stage in adjacent, but independent regions [92,94]. Like in the zebrafish, defects in both the hematopoietic and angioblastic lineages are observed in embryos lacking VEGF-R2/Flk1 [95], but unlike in the zebrafish, in mouse embryos lacking the transcription factor Scl/Tal-1, mainly the HSPC production is affected and secondary capillary sprouting from blood vessels are perturbed [96,97,98]. Additionally, Indian hedgehog, one of the three mammalian hedgehog isoforms, signals from the extraembryonic endoderm to promote vascular and hematopoietic cells [99]. Notably, mouse embryos lacking hedgehog signaling experience regional defects in vasculogenesis, affecting the anterior (but not posterior) region of the dorsal aorta and to the vessels of the yolk sac. This phenotype can be reversed with VEGF treatment [100,101]. On the contrary, over-activation of Hh signaling increases the expression of the Notch ligand DLL4 in the vascular cells. The secreted growth factor VEGF-A is found in multiple isoforms in mammals and signals through multiple receptor tyrosine kinases, including VEGFR2/FLK1, FMS-like tyrosine 1 (VEGFR1/FLT1), FLT4 (VEGFR3) and NEUROPILIN (NP)1 and NP2, all of which are abundantly expressed in the vascular system. Downstream of this cascade, the vascular cord segregates based on its identity.

### 6.2. Notch Signaling in Arterial Specification

Surprisingly, endothelial cells of the dorsal aorta lack expression of most arterial markers, including ephrinB2-LacZ, Cx40, Hey2, Nrp1, Notch1, Notch4, or Jag1 before E8.25 and only show weak expression of Cx37 and Hey1. Dll4 was the only robustly expressed (arterial) marker at this stage [102]. Just after this period, the embryo acquires blood circulation. Blood flow itself is essential to maintain of arterial identity since mouse embryos lacking the cardiac sodium–calcium ion exchanger Ncx1 do not develop blood flow, which results in inhibition of endothelial Notch activation and expression of EphrinB2 in the DA [103]. Finally, the Forkhead box c proteins Foxc1 and 2 can also induce DLL4 expression in the arterial endothelium. Notably, the promoter of the DLL4 gene harbors Foxc-binding sites and, thus, foxc appears to positively regulate Notch signaling by activating the Dll4 promoter during arterial specification [104]. Arterial, but not venous vessels co-express Notch1, Notch4, Jag1, Jag2 and Dll4 [31], whereas venous endothelium is identified by the expression of Coup-TFII and EphB4 like in zebrafish. However, the individual requirements of these receptors and ligands are still unclear.

### 6.3. Genetic Knockout Mice of Notch Signaling and Aorta Specification

Notch1 knockout mice show a reduction in the radius of axial vessels, cannot properly remodel the vasculature and die during embryonic development, while deletion of Notch4 has no major effect in vessel formation. However, Notch1/Notch4 double mutants show a more severe phenotype in abnormal axial vessel development than the Notch1 mutants alone, suggesting that the two genes are at least partially functionally overlapping [105]. Similarly, mice lacking even one copy of the Notch ligand Dll4 exhibit severe vascular defects [106]. The role of JAG1 and JAG2 in this context is more elusive. While JAG2 deletion has no impact on angiogenic development and HSPC formation, Jag1 knockout mice die between embryonic day E10.5–E11.5 due to aberrant angiogenesis in the yolk sac and embryonic vasculature, although the aorta is formed and expresses the key marker EfnB2 and CD44 [107,108]. During sprouting angiogenesis, the process whereby blood vessels create secondary capillaries, tip cell selection is controlled by an antagonistic role for DLL4 and JAG1 and fine balance of these two ligands [48].

## 7. Notch Activity Requirement for HSC Emergence in the Mouse Model

There is multiple evidence from different vertebrate animal models that indicate the requirement for Notch activity in HSC emergence during embryonic development. Data has been collected from different Notch loss-of-function mutants, but the interpretation of the results is not always straightforward.

### 7.1. Notch Receptor Mutants

Experiments with embryonic chimeras demonstrated that Notch1-deficient cells failed to contribute to hematopoiesis after E15.5 of development, indicating that Notch1 is a cell autonomously needed for definitive hematopoietic development [109,110]. Curiously, only the definitive wave is affected since the early yolk sac hematopoiesis is preserved in these embryos. This is the most elegant demonstration of the Notch requirement for definitive hematopoiesis, although the presence of HSCs or HE in AGM was not specifically tested and no conclusions can be drawn as to the temporal requirement of Notch1 in this process. Studies in both Notch1 and Rbpj knockout embryos have confirmed the lack of HSCs or pre-HSCs at early stages of AGM development, though arterial development is also affected [109,110]. In contrast, Notch2 knockouts show no obvious hematopoietic defects [110,111] and Notch3 and Notch4 knockouts are viable, without obvious defects in HSC generation [112,113]. 

### 7.2. Notch Target Genes

In both mice and zebrafish, Notch signaling functions genetically upstream of the key and most nascent hematopoietic transcription factor Runx1 [114,115]. Runx1 expression occurs in the dorsal aorta and the mesenchyme below [11,29]. Although Notch signaling activates several genes that are important for HSC formation, including Hes1, Hey2 and Gata2, there is no evidence for direct regulation of Runx1 by Notch. Instead, Notch contributes to the oscillatory expression of Hes1 and Gata2 and the latter drives Runx1 expression as part of a transcriptional complex with further unknown factors [46,116]. Accordingly, the hematopoietic defect of Notch signaling deficiency can be rescued by the induction of Runx1 [117]. Notch signaling itself participates in the transcriptional regulation of several Notch receptors and ligands by positive or negative feedback loop. Mutant RBPJ embryos which lack any Notch transcriptional activity show a specific reduction in the expression level of Jag1 and Jag2 in the AGM aortic cells, suggesting that Notch activity in the aorta can control the expression of these ligands [118]. Strikingly, Jag1 deficient AGMs specify the aortic endothelium, but there is no IAHC formation [119]. However, Jag1 knockout AGM does possess cKIT along the dorsal aorta [120]. They retain high expression of endothelial genes, do not gain CD45 expression and show reduced CFU-S activity [120]. The Jag1 knockout aorta also almost completely lacks Runx1 expression and has no Gata2 expression [119]. Next to Gata2 activation, Notch signaling also initiates the expression of the transcriptional repressor Hes1 in the dorsal aorta. Curiously, Hes1/Hes5 double knockout AGM (Hes5 compensates for Hes1 loss), present a striking phenotype, whereby IAHC formation occurs, but the cells within the IAHC have no stem cell activity upon transplantation into recipients. Hes1/5 mutant AGMs have increased levels of Gata2 (since the negative feedback loop is compromised) and this increase can be reduced to WT levels with the compound DAPT upon which the HSPC activity is partially restored [116]. How the initiation of Jag1 expression fits in this Notch mediated control of HSC emergence is currently unknown, although the HSC loss in Jag1 mutants can be overcome with ectopic Gata2 expression.

### 7.3. Repressors of Notch Activity

The Sox transcription factor Sox17 can directly counteract Notch1 expression by binding to its promoter or the promoter of Dll4 and Notch1 and thereby modulate Notch activity [121]. Genetic loss of either Sox17 or Notch1 during EHT results in increased production of hematopoietic cells due to loss of Sox17-mediated repression of runx1 and gata2 [121]. Studies using DAPT during in vitro culture of AGMs from mouse and chicken have also described an increase in hematopoietic progenitor numbers [121,122]. At least in the mouse study, the authors determined that this increase in number was due to proliferation of the progenitors and not EHT as assessed by BrdU labeling [121]. However, the increase in EHT can be abolished by increased Notch signaling [121].

Altogether, the findings on Notch participation in HSC emergence suggest that it is essential for the formation of HSC activity. The ligand JAG1 is crucial for HSC generation, while the right activation of the downstream targets, especially Hes1 is necessary for HSC activity. Hes1/5 double mutants have plenty of IAHC, but no HSC activity.

## 8. Notch Activity during In Vitro Differentiation of Embryonic Stem Cell to Blood

Mouse Embryonic stem (mES) cells are pluripotent cells derived from the inner cell mass of blastocyst-stage embryos and have the capability to differentiate into progeny of the different germ layers in culture [123,124]. It provides a powerful model system for studying mammalian development. More recently, human ES (hES) cell cultures have also been established although they differ in their properties. Notably, mES cells do not differentiate to trophectoderm in culture, but hES cells can be induced with BMP4 upon which they will give rise to cells that display characteristics of the trophoblast lineage [125]. Under appropriate culture conditions, mostly by removing the stem cell maintenance factor LIF, mES cells can generate cells in vitro that express the hematopoietic/vascular marker Flk-1 [126], together with mesodermal gene *Brachyury* [127] in some specific conditions. These cells can give rise to vascular smooth muscle (VSM) cells, in addition to hematopoietic and endothelial progeny. Based on these observations, it was postulated that these cells are equivalent to the yolk sac hemangioblast and, to date, there is no standardized protocol available to derive multi-lineage, long term repopulating HSC from them [128,129]. Like during embryonic development, key transcription factors leading to vascular and hematopoietic transition, including Scl/Tal1 and Runx1, have been also found to be essential during ES cell differentiation to blood [130,131]. Interestingly, Scl-/- deficiency in endothelial cells leads to a growth deficiency in monolayer cultures, but can be partially reverted by culturing them in 3-dimensional aggregates [131]. However, like their embryonic counterpart, blood generation is completely diminished and only the smooth vascular muscle is retained [131]. More recently, efforts have been made to induce the generation of hematopoietic cells beyond the yolk sack stage, i.e., lymphoid cells and transplantable HSC. Co-culture with Notch ligands and culture media with defined cytokines have improved these efforts, but has not been accomplished yet [132,133]. Several studies have therefore focused on understanding and resolving the need for Notch activity during in vitro differentiation. The repertoire of Notch signaling molecules was assessed during the time course of human ES cell differentiation and was mainly found to be expressing the Notch receptor Notch4 and the ligands Dll4 and Jag2 [134]. Interestingly, they found the ligand Dll4 to be expressed at higher levels on endothelial fated cells and the expression level was declined upon transition to the hematopoietic fate [134]. Several studies have explored the Notch pathway involvement during hematopoietic differentiation of mouse and human ES. Notch activation increases the frequency of CD45+ blood cells [126,134,135,136], while NOTCH inhibition with DAPT decreases the percentage of CD45+ cells in cultures of hES derived CD34^+^CD73^−^CD43^−^ progenitors [137]. NOTCH activation in hPSC cultures is predominantly mediated through the NOTCH ligand, DLL4, expressed by endothelial cells [134]. More recent studies have focused on whether arterial specification is a prerequisite for enhanced lymphoid and HSC (like) fate. Human ES cultured in chemically defined conditions and isolated HE cells were either exposed to DLL4 to activate Notch, or DAPT to inhibit Notch during EHT. After this Notch manipulation, hematopoietic cells were assessed for the number of colonies and type of blood cells they could generate [138]. In this process, the authors discovered that NOTCH activation (through DLL1 exposure) in hPSC-derived immature HE progenitors lead to the formation of CD144^+^CD43^−^CD73^−^DLL4^+^Runx1^+^ hemogenic arterial-like endothelial cells, which requires NOTCH activation to undergo EHT and produce definitive lympho-myeloid and erythroid [138]. This is in contrast with the previous report in which they specifically found that HE cells were DLL4^−^ and distinguished from the arterial precursors [137]. Strikingly, DLL4^+^ HE could only produce blood cells when induced with OP9-DLL4, but not if they were exposed to DLL1 [138].

In summary, HE activity was associated with DLL4^+^ cells in studies employing ES differentiation. They further highlight that Notch manipulation, especially its activation during HE to EHT can enhance blood progenitor production.

## 9. Is Arterial Specification Necessary for HSPC/HSC Emergence?

**Figure 3:**
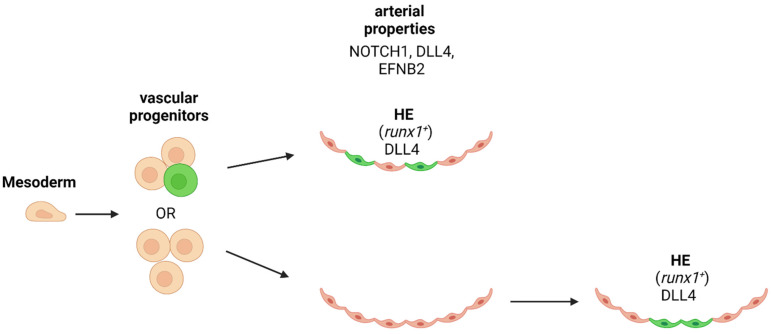
Concepts of hemogenic endothelium specification. Hematopoietic potential is highlighted in green. Early mesodermal vascular progenitors might contain hemangioblasts, a bipotential cell with endothelial and hematopoietic potential already, or hematopoietic potential is acquired secondary after commitment to a vascular arterial fate. Top: early vascular progenitor cells have a rare population of cells that are hemogenic. bottom: hemogenic endothelial cells are specified after arterialization of the endothelium. HE: hemogenic endothelium. Green: vascular cell with hematopoietic properties. Created with BioRender.com (accessed on 15 January 2022).

The genetic program between endothelial and hematopoietic cell types and fate tracing studies are unequivocally supporting the common origin of both cell types. If the rare population of HE is already determined before the vascular progenitor migrates to the midline of the embryo to form the axial vessels, or only after the segregation of the vascular cells into arteries and veins, is under debate (Figure 3). Since HSCs are generated in the aortic niche of the AGM, it is likely that arterial specification is a pre-requirement for the hemogenic precursor of HSPCs (Figure 3). One of the key factors in defining this requirement is the Notch activity and the expression of ligands and target genes. This question has been addressed by different groups in several experimental models: ES cell differentiation, mouse embryos and zebrafish embryos.

In zebrafish morphants for Etsrp or Scl/Tal1 that have an earlier block in endothelial development (before the onset of angioblast migration) than Hh-, Vegf- or Hey2-depleted embryos could not be rescued for their *runx1* deficiency through Nicd activation [81]. These findings suggest that early endothelial programming and arterial priming is an obligate prerequisite HSPC formation in the aorta. In order to address whether arterial requirement is a prerequisite for HE development, Bonkhofer et al. separated the dorsal aortic cells based on *runx1* reporter gene expression levels and then were subjected to transcriptomics. They found arterial gene expression (Dll4) present in the Runx1+ fraction. Furthermore, they propose that Runx1 downregulates the arterial fate of HE cells as they undergo EHT [139].

**Figure 4:**
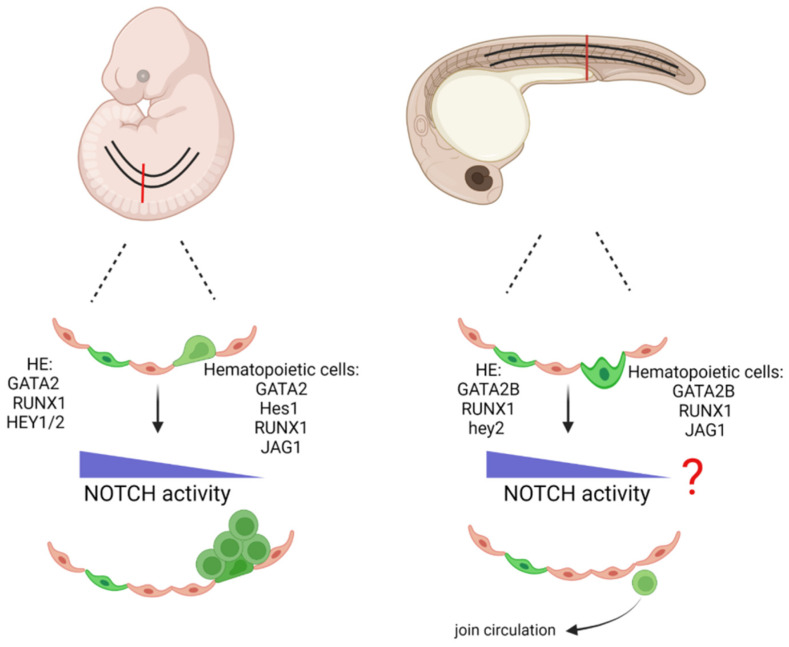
A comparison of zebrafish and mouse embryonic aortic hematopoiesis. Depicted are Notch signaling molecules that act during hemogenic endothelium specification and intra-aortic cluster formation. In mouse EHT, the hematopoietic cells accumulate as clusters and have low(er) Notch activity (left). During zebrafish hematopoiesis, cells undergoing EHT leave the aorta individually and do not accumulate as clusters. Green: cells with hematopoietic properties. HE: hemogenic endothelium. Created with BioRender.com (accessed on 15 January 2022).

To investigate whether arterial specification was mandatory for downstream HE/IAHC formation, the para-splanchnopleura (the precursor tissue of the AGM) from E9-9,5 mouse embryos that lack Efnb2 (the KO die by E11.5) was cultured ex vivo and examined for hematopoietic potential compared to wild type embryos. The analysis found that Efnb2 knock out para-splanchnopleura cannot generate blood ex vivo. Interestingly, the EfnB2 KO vasculature have comparable levels of the arterial- associated genes such as Notch4, Dll4 and Hey2, but abnormally low levels of hemangioblast/hematopoietic genes, including Scl/Tal-1, Runx1 and Foxc2 [140]. They hypothesize that the interaction between EFNB2 and EFNB4 during early angiogenesis is important for HE development [140]. These two reports provide some experimental evidence for a requirement of an arterial fate before a HSPC competent HE is established. Whether or not full arterial specification is needed remains unknown since all studies focus on very few (distinct) genes and expression, but do not assess the extent of arterialization of HE. Elegant experiments using two types of Notch activity reporter revealed that arterial cells experience high Notch activity during their ontology. In contrast, HE and IAHC have only been subject to low NOTCH activation throughout development [120]. Further supporting this notion, treatment of AGM explants of mouse and chicken (performed after E9.5 for mouse) with DAPT, increases hematopoietic output in culture [121,122]. The opposite was also detected in the chicken AGM explants. Here, more hematopoietic progenitors were observed with DAPT, when the colony forming units were assessed [122]. These experiments highlight the developmental time and duration sensitivity of Notch activity. Furthermore, mouse HSPC form clusters of cells that “hibernate” within the aorta and supposedly mature into HSCs. In the zebrafish model, this behavior is not present. Instead, the HSPC seems to emerge in Notch activated state and quickly join circulation without forming clusters of cells within the aorta (Figure 4) [89]. Thus, it is tempting to speculate that Notch activity or quality changes within this window of time. In line with this speculation, some cells in the IAHC gain NOTCH2 expression and Immunophenotyping of cKIT positive clusters for DLL4 expression shows a decrease in its level that is anti-correlated to the size of the cluster [38].

Finally, plasticity of ES-derived hemogenic cells may be totally different from the embryonic precursors. One of the studies separated arterial endothelium from HSPC competent HE by using cell surface markers CD73^−^CD184^−^ during hES differentiation to blood. When Day 8 Embryoid Bodies are separated based on CD34, CD73 and CD184, they find that arterial cells expressing EfnB2 are highly enriched in the CD73medCD184+ cells, but EHT competent HE cells are found in the CD34^+^CD73^−^CD184^−^DLL4^−^ cell compartment based on hematopoietic marker [137].

On the other hand, a study overexpressing ETS1 or modulating MAPK/ERK signaling pathway at the mesodermal stage of hES differentiation to blood induced arterial type HE with DLL4^+^CXCR4^+/−^ phenotype that dramatically enhanced the lymphoid potential of the HE by more than 100-fold [141]. It is more than likely that lymphoid potential is dependent on Notch activity and arterial fate of HE. Perhaps some specific Notch activation in the HE/IAHC is needed to specify IAHC that can contribute to all lineages, including lymphoid cells.

## 10. How Much Notch Activity Is Needed for HSCs?

The studies presented and discussed in this review support a critical role for Notch signaling activity in HPC emergence and HSC activity. We are yet to determine if developing hematopoietic progenitors and HSCs in IAHC/aorta differ in their requirements of Notch activity (receptor-ligand interaction, or different downstream targets).

In this regard, we are still unable to properly translate these findings into in vitro settings and to generate transplantation competent HSCs. Reports where Notch signaling has been manipulated during hematopoietic development by either lowering the levels with DAPT or activating it by exposing the cells of interest to Notch ligands have not been able to accurately mimic the in vivo conditions. Hence, it is only plausible that we are still missing some vital knowledge or components about the process. The studies in search for a role in arterial specification as a requirement for HSPC emergence did not test the identified precursor cells for stem cell activity [134,137,138,139,141]. During embryonic HSPC emergence from the dorsal aorta, a complex array of Notch ligands, receptors and direct downstream targets are involved. It is likely that different numbers of receptors, or ligands or combinations of receptors or ligands are unique to arterial or HE/IAHC cells. Clues for this difference can be found in the large quantity of single cell RNA sequencing data that is available for AGM derived hematopoiesis. Several Notch receptors, ligands and downstream targets including Notch1, Notch4, Dll4, Jag1, Hey2, Hey1 and Hes1 have been reported in the HE population. Here, we propose that Notch is required at several stages of HSPC formation. Early arterial cells express Notch target genes and downstream Jag1 expression is critical for HE/EHT and IAHC formation of all blood cells from the dorsal aorta. Notch activity also seems to dictate HSC activity. Here, it is essential that Hes1 and Gata2 are expressed in an oscillatory manner. Recent studies in the neuronal stem cell compartment studied the oscillation of Hes1 and its effect on stem cell activity [142]. Strikingly, they suggest that Notch1-induced Hes1 oscillation is a cue for cell proliferation and a transition from quiescent to active states of neuronal stem cells.

Importantly, the type of Notch activity changes over time. This might be the challenging aspect to reproduce in vitro by modulating all pathways (and cells) with inhibitors like DAPT/y-secretase that abolish all Notch activity, or by activating Notch activity with one type of ligand. The use of ligand or receptor specific inhibitors or activators would be ideal to assess the true impact of each Notch signaling molecule during hematopoietic/HSC development. As an example, the use of a blocking antibody directed against DLL4 during AGM hematopoiesis has provided great insight; blocking DLL4 during IAHC formation greatly enhances IAHC size and to a lesser extent HSC activity [38]. Finally, perhaps we will be able to decipher the molecular programs that are driven by Notch to specify HSCs by mining the vast number of single cell RNA sequencing data [29,33,37,143,144,145,146,147,148,149]. In this case, we can circumvent modulation of Notch activity and directly induce the desired downstream signaling.

## Data Availability

Not applicable.
